# Developing Smart Nanoparticles Responsive to the Tumor Micro-Environment for Enhanced Synergism of Thermo-Chemotherapy With PA/MR Bimodal Imaging

**DOI:** 10.3389/fbioe.2022.799610

**Published:** 2022-02-21

**Authors:** Mingfang Luo, Yijie Lv, Xunrong Luo, Qingfa Ren, Zhenbo Sun, Tianping Li, Ailing Wang, Yan Liu, Caixia Yang, Xianglin Li

**Affiliations:** ^1^ School of Medical Imaging, Binzhou Medical University, Yantai, China; ^2^ Department of Radiology, Affiliated Hospital of Qingdao University, Qingdao, China; ^3^ Department of Clinical Laboratory, Yantai Affiliated Hospital of Binzhou Medical University, Yantai, China

**Keywords:** magnetic hyperthermia, chemotherapy, magnetic resonance imaging, photoacoustic imaging, theranostics

## Abstract

With the development of nanotechnology, a theranostics nanoplatform can have broad applications in multimodal image-guided combination treatment in cancer precision medicine. To overcome the limitations of a single diagnostic imaging mode and a single chemotherapeutic approach, we intend to combat tumor growth and provide therapeutic interventions by integrating multimodal imaging capabilities and effective combination therapies on an advanced platform. So, we have constructed IO@MnO_2_@DOX (IMD) hybrid nanoparticles composed of superparamagnetic iron oxide (IO), manganese dioxide (MnO_2_), and doxorubicin (DOX). The nano-platform could achieve efficient T2-T1 magnetic resonance (MR) imaging, switchable photoacoustic (PA) imaging, and tumor microenvironment (TME)-responsive DOX release and achieve enhanced synergism of magnetic hyperthermia and chemotherapy with PA/MR bimodal imaging. The results show that IMD has excellent heating properties when exposed to an alternating magnetic field (AMF). Therefore, it can be used as an inducer for tumor synergism therapy with chemotherapy and hyperthermia. In the TME, the IMD nanoparticle was degraded, accompanied by DOX release. Moreover, *in vivo* experimental results show that the smart nanoparticles had excellent T2-T1 MR and PA imaging capabilities and an excellent synergistic effect of magnetic hyperthermia and chemotherapy. IMD nanoparticles could significantly inhibit tumor growth in tumor-bearing mice with negligible side effects. In conclusion, smart IMD nanoparticles have the potential for tumor diagnosis and growth inhibition as integrated diagnostic nanoprobes.

## 1 Introduction

Chemotherapy is one of the primary treatment methods for cancer. However, it still has the limitations of multidrug resistance, multiple side effects, and low drug bioavailability (Du et al., 2012). Recently, research of synergistic therapies through different collaborative strategies has received increased attention (Gu et al., 2019). Various efforts have been made to develop multifunctional nanoplatforms that can integrate chemotherapy with other therapeutic strategies, such as hyperthermia, photodynamic therapy, or immunotherapy, which could achieve synergistic effects and overcome the shortcomings of chemotherapy (Liu et al., 2015; Chen et al., 2017; Liu et al., 2017; Lohitesh et al., 2018; Luo et al., 2018; Mai et al., 2019). Among the numerous strategies, magnetic hyperthermia has been commonly applied in cancer treatment research because of its noninvasive nature, accurate targeting, high penetration depth, and good therapeutic effect. Magnetic hyperthermia usually selects magnetic nanoparticles as the thermal medium. After selectively allowing magnetic nanoparticles to enter the tumor and then exposing the tumor to the external alternating magnetic field (AMF), the internalized magnetic nanoparticles generate heat, which increases the internal temperature of the tumor. In general, increasing the temperature of the tumor tissue (>43°C) can effectively kill tumor cells (Hervault and Thanh 2014; Mantso et al., 2016). Moreover, hyperthermia not only has a direct effect on cancer cells but also damages the stability of cancer cells, making them more susceptible to chemotherapy or radiotherapy (van der Zee 2002). Therefore, magnetic hyperthermia based on magnetic nanoparticles has emerged for collaborative chemotherapy, radiotherapy, or gene therapy (Liu et al., 2020).

It is worth noting that accurate diagnosis is another essential component of cancer prognosis. Therefore, integrating multiple imaging and treatment functions on one platform has been considered a promising method to optimize treatment plans, and improve the effectiveness of treatment (Li et al., 2020). It has been reported that the superparamagnetic iron oxide magnetic nanoparticles (SPIO) are commonly used as T2 MR imaging contrast agents and magnetic hyperthermia therapy agents (Huang et al., 2014). For instance, Du et al. (Du et al., 2019) developed an SPIO-based diagnosis and treatment nanoparticle to achieve multimodal MR/magnetic particle imaging for improved magnetic hyperthermia. However, because of the tumor heterogeneity, a simple T2 MR imaging contrast agent cannot effectively distinguish the conditions within the lesion, tumors, and some low-intensity areas, which may easily lead to misdiagnosis. Therefore, developing a specific T2-T1 MR imaging nanoplatform is critical to achieving an accurate diagnosis (Lu et al., 2018). Since MR imaging is relatively expensive and unsuitable for continuous imaging over long periods (Zhao et al., 2014), compared with other imaging methods, PA imaging provides high-resolution images with low cost, deep penetration depth, and rapid scanning speed (Fu et al., 2015; Yang et al., 2018). By integrating different imaging methods, the inherent drawbacks of a single imaging method can be avoided.

TME is usually characterized by hypoxia, weak acidity environment, excessive glutathione (GSH), and hydrogen peroxide (H_2_O_2_), which seriously impedes the effective treatment of tumors (Xu et al., 2019; Guo et al., 2020). In recent years, it has been found that MnO_2_ has the characteristics of good biocompatibility, capable of carrying the drug, tunable structures, and reversible TME, so that it has a variety of functional applications, including bioimaging, drug-loading carrier, biosensing, and tumor therapy (Ding et al., 2020; Zhang and Ji 2020). For instance, MnO_2_ can react with H^+^, H_2_O_2_, or/and GSH to produce Mn^2+^, O_2_, or/and oxidized glutathione to reverse the TME. Furthermore, the Mn^2+^ generated during this process can be used to T1 MR imaging contrast (Jing et al., 2019). Moreover, more imaging abilities about MnO_2_ also has been found. Such as PA imaging, photothermal imaging, and ultrasonic imaging (Gao et al., 2017; Lin et al., 2019; Wang et al., 2019). It is worth noting that Liu et al. (2018) (Liu et al., 2018) have shown that MnO_2_ could also achieve switchable PA imaging based on the TME response. This study has opened new avenues for MnO_2_-based multimodal and multiparametric cancer imaging and diagnosis.

Here, we have developed smart nanoparticles (IMD) for MR/PA imaging-guided synergism of magnetic hyperthermia and chemotherapy. The superparamagnetic iron oxide (IO) nanoparticles with good magnetic hyperthermia properties were obtained by thermal decomposition. Then MnO_2_ nanosheets were grown around IO. Finally, the chemotherapeutic drug DOX was absorbed onto the surface of IO@MnO_2_, as depicted in Schematic 1. The high level of GSH in the acidic TME could promote IMD degradation, resulting in the controlled release of DOX at the tumor site. In addition, IO has an excellent T2 MR imaging contrast effect resulting in special MR imaging properties. Manganese dioxide is a potential contrast agent for PA imaging, and the released Mn^2+^ is a good candidate for the T1 MR imaging. *In vitro* and *in vivo* antitumor therapy showed that the smart nanoparticles could effectively eliminate tumors by synergistic therapy using T2-T1 MR imaging and PA imaging, and this offers great potential for future clinical applications.

**FIGURE 1:**
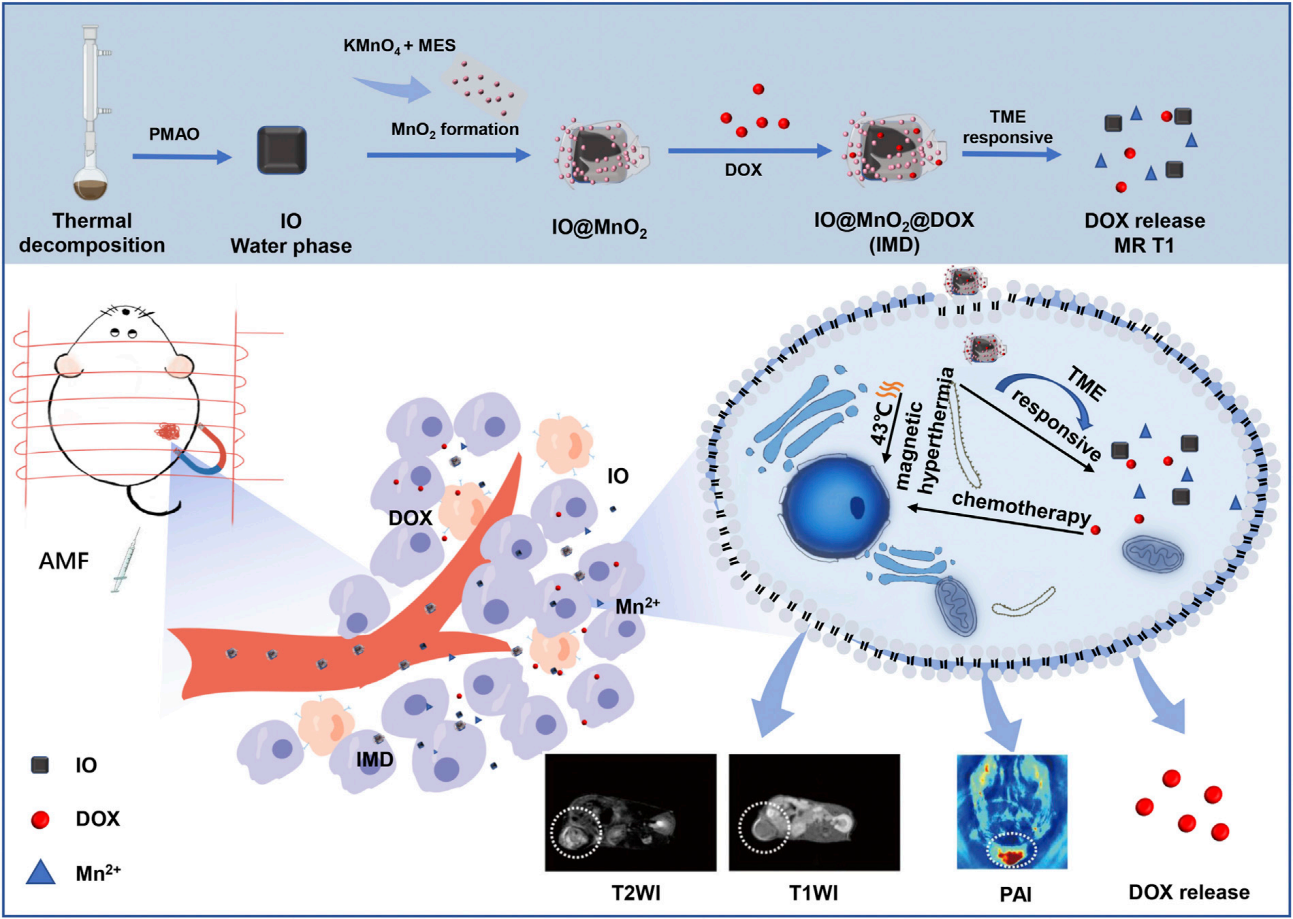
Schematic illustration of the intracellular behavior of IMD for MR/PA imaging-guided magnetic hyperthermia and chemotherapy.

## 2 Materials and Methods

### 2.1 Chemicals and Materials

All the chemical reagents were of analytical grade and were used without additional purification. Iron (III) acetylacetonate (99%), squalene (98%), dibenzyl ether (99%), decanoic acid (99%), 2-(N-Morpholino)ethanesulfonic acid (MES) (99%), MTT Formazan powder, and Doxorubicin hydrochloride (DOX) (98%) were purchased from Shanghai Aladdin Biochemical Technology Co., Ltd. (Shanghai, China). KMnO_4_ and poly (maleic anhydride-alt-1-octadecene) (PMAO, MW = 30,000–50,000 g mol^−1^) were purchased from Sigma-Aldrich (St. Louis, MO, United States). Fetal bovine serum was obtained from AusgeneX Pty Ltd. (Gold Coast, Australia). RPMI-1640, penicillin, and streptomycin were obtained from HyClone (GE, United States). Ultrapure water (18.2 mΩ cm^−1^) generated using a Millipore Milli-Q purification system.

### 2.2 Synthesis of Iron Oxide

The IO was synthesized based on a thermal decomposition method (Guardia et al., 2012; Guardia et al., 2014; Materia et al., 2015). Briefly, in a 100 ml two-neck round-bottom flask containing 18 ml dibenzyl ether, 0.353 g (1 mmol) of iron (III) acetylacetonate, 0.69 g (4 mmol) of decanoic acid, and 7 ml of squalene were added. After degassing at 65°C for 120 min, the mixed solution was heated to 200°C (3°C/min) and stored at this temperature for 2.5 h. Finally, the solution was heated to the reflux temperature at a heating rate of 7°C/min and kept at this temperature for 1 h. When the solution cooled down to room temperature, the products were washed using a 4-fold volume of acetone and chloroform successively and centrifuged at 8,000 rpm. To transfer the IO in water, a previously reported method was used (Moros et al., 2010). First, PMAO (250 mg) and chloroform (100 ml) were put into the flask. After dissolving the polymer with magnetic stirring, IO (20 mg) were added to the solution. The mixture was stirred overnight at room temperature. Then, the solvent was removed in a vacuum by rotary evaporation, and after that chloroform (2 ml) was added. The products were suspended in NaOH (0.05 M; 20 ml), stirred at 25°C to accelerate the complete evaporation of chloroform until the solution turned brownish-black. Finally, the obtained water-soluble IO was freeze-dried and weighed to obtain 18.67 mg water-soluble IO for subsequent experiments.

### 2.3 Synthesis of IMD

The IO@MnO_2_ (IM) nanocomposites were obtained by a mild ultrasonication method (Deng et al., 2011; Zhang et al., 2014). First, IO water solution (10 ml; 1.6 mg/ml), KMnO_4_ aqueous solution (10 ml; 10 mM), and MES buffer (300 μL, 0.1 M, pH = 6.0) were mixed in a 50 ml centrifuge tube and sonicated for 30 min until a brownish-black product was formed. Subsequently, the product was washed three times with deionized water, and the final product was resuspended in deionized water (10 ml). Then an aqueous solution of DOX (2 mg) was added to the former solution and shaken for 12 h in darkness at 25°C. After centrifugation (12,000 rpm, 20 min), the product was dispersed in deionized water and stored at 4°C in darkness.

### 2.4 Characterization of the IMD

The morphology and microstructure of the IO, IMD, and GSH-reduced (pH 6.8, GSH 200 μM) IMD were observed using a transmission electron microscope (JEM-1400, JEOL, Japan). High-resolution transmission electron microscopy (HRTEM) (FEI, TF20, United States), equipped with an energy dispersive spectroscopy (EDS) attachment, was used to observe IMD and obtain the selected area electron diffraction (SAED) pattern and EDS of IMD. The size distribution and Zeta (*ζ*) potentials were measured using the Zetasizer Nano ZS (Malvern, England) at 25°C. The IMD UV-vis spectra and DOX content were determined by a UV-vis spectrometer (TU 1900, Persee, China), and the wavelength ranged from 300 to 900 nm. IMD treated with different GSH fluorescence emission spectra were monitored by a fluorescence spectrophotometer (F-7000, Hitachi). The contents of iron and manganese in IM were measured by inductively couple plasma mass spectrometry (ICP-MS) (Agilent 7900, United States). The X-ray diffraction (XRD) patterns of IO and IMD were obtained using Cu-Kα radiation (*λ* = 1.543 A) on an X-ray diffractometer D8 advance (Bruker, Germany).

### 2.5 Magnetic Hyperthermia Performance

The magnetic hyperthermia performance and magnetic hyperthermia stability of IMD were evaluated. First, different concentrations of IMD ([IO]: 0, 0.2, 0.4, and 0.8 mg/ml) were loaded into a 2 ml centrifuge tube and then placed in a magnetically induced heating device (Shuangping SPG, China) with a frequency of 474 kHz and a current of 34.7 A for 600 s. The temperature increase process was recorded in real-time with an infrared thermal imaging camera (A315, FLIR, United States), and the temperature was recorded every minute. Magnetic hyperthermia stability was carried out alternately by heating for 5 min and cooling for 5 min.

### 2.6 Drug Loading and Release

The supernatant was collected during the synthesis of IMD. The absorbance of the supernatant at 482 nm was measured using a UV-Vis absorption spectrometer (TU 1900, Persee, China), and the content of free DOX was calculated according to the DOX standard curve. The encapsulation capacity and drug loading capacity of DOX in IMD was calculated according to the following equation (Xie et al., 2020):

$$\mathrm{Encapsulation capacity}=\;\frac{\mathrm{The weight of DOX in IMD}}{\mathrm{The total weight of DOX}}\times 100\%$$

$$\mathrm{Drug}\;\mathrm{loading}=\;\frac{\mathrm{The}\;\mathrm{weight}\;\mathrm{of}\;\mathrm{DOX}\;\mathrm{in}\;\mathrm{IMD}}{\mathrm{The}\;\mathrm{total}\;\mathrm{weight}\;\mathrm{of}\;\mathrm{IMD}}\times 100\%$$

The drug release was evaluated at 37°C: 1) pH 7.4, 2) pH 6.8 containing 2 mM GSH. The UV-Vis absorption of DOX was then measured to evaluate the drug release.

### 2.7 Fluorescence Recovery of IMD

To demonstrate the fluorescence quenching of manganese dioxide, different concentrations of GSH (0, 5, 10, 12.5, 25, 50, 100, and 200 μM) were mixed with IMD (IO concentration of 0.16 mg/ml) solution for 12 min. The emission spectrums of DOX (λ_ex_ = 593 nm) were recorded.

### 2.8 Evaluation of MR/PA Imaging Capacity of the IMD

The IMD dispersions (in PBS or GSH solution) with different concentrations (Fe: 0–0.093 mM) or Mn: 0–0.106 mM) were loaded into centrifuge tubes (250 μL) and examined by using a 7.0 T MR imaging scanner (Biospec 70/20, Bruker, Germany). The images were acquired using fast spin-echo sequences (Yang et al., 2018). T2-weighted imaging parameters: repetition time (TR) = 2,500 ms, echo time (TE) = 30 ms, acquisition matrix = 256 × 256 mm, and slice thickness = 1 mm. T1-weighted imaging parameters were TR/TE = 500/18 ms. T2 value measurement was carried out using an MSME-sequence (Multi-Slice Multi Echo), and T1 measurement was carried out using a T1 inversion recovery fast spin-echo (FSE) sequence (T1_map_RARE). MSME-sequence parameters: TR of 2,500 ms, 10 TE of 20, 40, 60, 80, 100, 120, 140, 160, 180, and 200 ms. T1_map_RARE parameters: TE = 8.5 ms, TR = 372.833, 400, 800, 1,500, 3,000, and 5,500 ms (Sun et al., 2020). The T1/T2 relaxation data value was calculated by selecting the same region of interest level in each sample using the ParaVision 6.0.1 tools provided by Bruker. The T1/T2 relaxivity (r1/r2) of IMD was obtained through the curve fitting of 1/T1 or 1/T2 relaxation time (s^−1^) vs. the Fe concentration (mM Fe) or Mn concentration (mM Mn) (Frazier et al., 2016).

To evaluate the performance of switchable PA imaging, IMD with different IO concentrations (0, 0.05, 0.1, 0.2, 0.4, and 0.8 mg/ml) were suspended in DI water. A multispectral optoacoustic tomographic (MSOT) imaging system (inVision 128-TF, iThera Medical, Germany) was used to image the phantom tubes (filled with 100 μL IMD). Additionally, to evaluate the effect of GSH on PA signals of IMD, different concentrations of GSH (100 μL; 0, 0.05, 0.25, 0.5, and 1 mg/ml) were mixed with 100 μL of IMD ([IO]: 0.8 mg/ml). The mixture was stirred for 12 min to ensure a complete reaction. All the PA data is obtained through the following parameters: excitation wavelengths are 680, 700, 750, 800, 850, and 900 nm, frame-rate of 10 Hz, a laser with a pulse duration of 10 ns, repetition rate of 10 Hz, 100 frames, step length is 0.2 mm, and the image post-processing is analyzed by delimiting regions of interest (ROI) on MSOT imaging software (Ho et al., 2014; Liu et al., 2018).

### 2.9 Cell Culture

4T1 cell lines were obtained from the Shanghai Cell Bank, Chinese Academy of Sciences (Shanghai, China). RPMI-1640 (used for cell growth) contained fetal bovine serum (FBS; 10%) and penicillin-streptomycin (1%) solution under a humidified atmosphere containing 5% CO_2_ at 37°C.

### 2.10 Cellular Uptake and Cytotoxicity Assay

The fluorescence imaging and Perls’ staining (neutral red method) were adopted to observe the cellular uptake of the IMD. First, 4T1 cells (2 × 10^5^ cells/well) were seeded in a confocal dish or 6-well plates and cultured overnight in RPMI-1640 medium at 37°C with 5% CO_2_. Then, 4T1 cells were incubated with PBS, IMD, or IMD with magnetic targeting (containing DOX 20 μg/ml) at 37°C (5% CO_2_) for 6 h. Alternatively, IMD at different concentrations ([IO]: 0, 0.2, 0.4 mg/ml) were added to cells and incubated for 6 h. Finally, the cells were stained with DAPI or Prussian blue/neutral red. An inverted microscope (XDS-3, Optika, Italy) or confocal microscope (TCS SP8, Leica, Germany) were used to obtain the data.

The cytotoxicity of DOX, IM, and IMD was investigated using an MTT assay. Briefly, 4T1 cells were transferred to 96-well plates with a density of 8 × 10^3^ cells/well and incubated overnight. Afterwards, DOX, IM, or IMD (100 μL) of different concentrations were added to each well. After incubation for 12 h or 24 h, the drug-containing medium was discarded, replaced with RPMI-1640 containing MTT (10 μL; 5 mg/ml), and further incubated for 4 h. Finally, the media was removed, and DMSO (100 μL) was added to each well. The absorbance was measured under a micro reader (Spark, Tecan, Switzerland) at 570 nm.

### 2.11 *In vitro* Synergistic Therapy Evaluation

To evaluate the synergistic effect of *in vitro* magnetic hyperthermia combined with chemotherapy, 4T1 cells (density of 1 × 10^5^ cells/well) were seeded on 30 mm well culture dishes and incubated overnight at 37°C with 5% CO_2_. After, the cells were incubated with an equivalent IO concentration and DOX concentration for 12 h. During this time, a magnet was placed under the culture dishes for 4 h to enhance the internalized of nanoparticles. The AMF only, IM with AMF and IMD with AMF groups were exposed to AMF for 20 min (frequency 474 kHz, current 34.7 A), and the cell survival rate (%) was investigated using the MTT assay.

### 2.12 Animal Models

Female BALB/c mice (5–6 weeks) were obtained from Jinan Pengyue Laboratory Animal Co. Ltd. (Jinan, China). All the animal experiments followed an approved protocol by the Binzhou Medical University Animal Ethics Committee. To establish 4T1 tumor models, about 8 × 10^6^ 4T1 cells in PBS (100 μL) were subcutaneously injected into the right hind limbs of each BALB/c mice. The tumor-bearing mice were used for the subsequent experiment when the tumor volume reached about 100 mm^3^.

### 2.13 *In vivo* MR/PA Imaging

To perform the MR/PA imaging, 4T1 tumor-bearing mice were intravenously injected with 100 μL of IMD (IO: 10 mg/kg) and then the MR scanner or MSOT imaging system was used to acquire the images at pre-designated time points (0, 2, 6, 12, and 24 h). The mice with PBS administration were used as control, and the magnetic targeting group was achieved by placing a magnet on the tumor for 4 h after the drug was administered.

### 2.14 *In vivo* Synergistic Therapy

When the tumor volume reached about 100 mm^3^, the 4T-1 tumor-bearing mice were randomly divided into groups as follows: 1) PBS, 2) IM, 3) DOX, 4) IM + AMF, and 5) IMD + AMF. Each group contained five mice and an intratumor injected with different formulations (100 μL) at a dose of IO 10 mg/kg and DOX 1 mg/kg body weight on days 0 and 7. The mice in group 4) and group 5) were exposed under AMF (frequency 474 kHz, current 34.7 A) for 20 min. After chemotherapy or magnetic hyperthermia treatment, the body weight and tumor volume of the mice were monitored every 2 days for 16 days. The tumor volume was calculated according to the equation (volume = length × width^2^/2). After therapy, one mouse was randomly sacrificed in each group, and their tumors were isolated and further stained with hematoxylin and eosin (H&E). After 16 days of treatment, all the remaining mice were sacrificed, and major organs (heart, liver, spleen, lung, and kidney) were retrieved for staining.

### 2.15 Statistics

Each experiment was repeated three times, and the results were expressed as mean ± standard deviation. The difference between groups was used the one-way ANOVA analysis, and when *p < 0.05, or **p < 0.01 were considered statistically significant.

## 3 Results and Discussion

### 3.1 Synthesis and Characterization of the Nanoparticles

IMD was prepared according to the method illustrated in Schematic 1. IMD was synthesized according to the method reported in the literature, and some modifications had been made (Guardia et al., 2012; Guardia et al., 2014; Materia et al., 2015). Firstly, the preparation of water-soluble IO (Fe_3_O_4_) via a regular two-step procedure that involved a thermal decomposition method and a solvent-exchange method. Then, KMnO_4_ was reduced by MES to obtain MnO_2_ nanosheet-coated IO in a simple mild ultrasonic method. Finally, the formation of IMD was accomplished by electrostatic adsorption of DOX onto IM. The successful synthesis and GSH-responsive degradation of IMD were demonstrated by measuring transmission electron microscopy (TEM) images and size distributions. Figure 1A shows that TEM images demonstrating that IO, IMD, and GSH-reduced IMD had unique morphologies. The shape of IO is approximately cubic, and in IMD TEM, we found that after reducing KMnO_4_ with MES at room temperature, the MnO_2_ nanosheets were successfully grown around IO. Its structure was consistent with the results of a previous study (Deng et al., 2011). Additionally, the nanoparticles were degraded, and the MnO_2_ structure could not be observed in the presence of H^+^ and GSH. Figure 1B shows the XRD results of IO and IMD, in which the diffraction peaks of IO and IMD are consistent with the Fe_3_O_4_ diffraction data (JCPDS Card No. 19–0629). The IO diffraction peaks are located at 30.1°, 35.54°, 37.1°, 43.26°, 53.48°, 57.08°, and 62.7°, which correspond to the (220), (311), (222), (400), (422), (511), and (440) planes of Fe_3_O_4_ respectively. In addition, diffraction peaks are observed at 2θ = 36.85° and 65.80° in IMD, which correspond to the phases of MnO_2_ (JCPDS Card No. 80-1098) (Zhang et al., 2014; Qiao et al., 2017). Figure 1C is the HRTEM image of IMD, where the measured lattice fringe spacing of IMD is 0.303 nm, corresponding to the crystal plane distance of (220) planes of Fe_3_O_4_. The SAED pattern (Figure 1D) is consistent with the XRD result. From the inside out, these polycrystalline rings point to the (220), (311), (400), (511), and (440) planes of Fe_3_O_4_ with the face-centered cubic phase. Besides, the EDS results (Figure 1E) also verified the composition of elements. These results indirectly indicate the crystal properties and successful synthesis of IMD. The DLS and Zeta potentials results show that the average hydrodynamic diameter of IO dispersed in deionized water was 23.19 ± 5.99 nm (PDI: 0.24), and the surface was negatively charged (−44.75 mV). The size and zeta potential of prepared IM was 119.33 ± 1.99 nm and −22.95 ± 0.84 mV, and this increased to 221.93 ± 19.36 nm and −18.75 ± 1.43 mV after loading with DOX (Figures 1F,G). These results indicated that IMD was successfully prepared, and it could be degraded in the presence of GSH. From the UV-vis spectrum (Figure 1H), we also confirmed the existence of DOX in the IMD, and the encapsulation capacity and drug loading capacity of DOX was 80 and 5.75%, respectively. ICP-MS confirmed that the loading capacity of IO and MnO_2_ in the IM were 61.06 and 38.94%, respectively. In the drug release experiment, we found that the release of DOX was at a low rate (7.86% in 36 h) at pH 7.4. Accelerated drug release (38.27% within 36 h) was detected when the pH dropped to 6.8 with GSH (2 mM) (Supplementary Figure S1).

**FIGURE 1:**
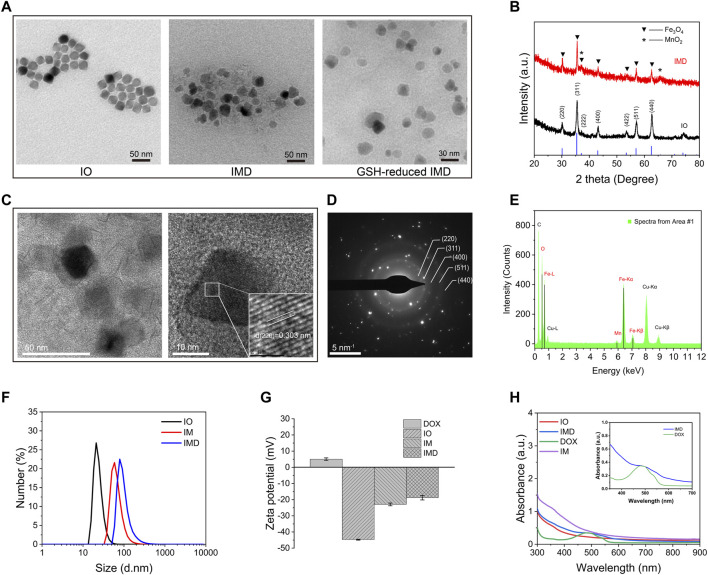
Characterization of different nanoparticles. **(A)** TEM imaging of IO, IMD, and GSH-reduced IMD. **(B)** XRD patterns of IO and IMD. **(C)** The HRTEM image, **(D)** SAED pattern, and **(E)** EDS analysis of IMD. **(F)** DLS and **(G)** Zeta potential of different nanoparticles. **(H)** The UV-visible spectrum of different nanoparticles with equivalent IO, MnO_2_, and DOX concentrations. ([DOX]: 16 μg/ml, [IO]: 0.16 mg/ml, and [MnO_2_]: 0.1 mg/ml). The inset shows the enlarged region from 350 to 700 nm of IMD and free DOX.

### 3.2 Magnetic Hyperthermia Performance

**FIGURE 2:**
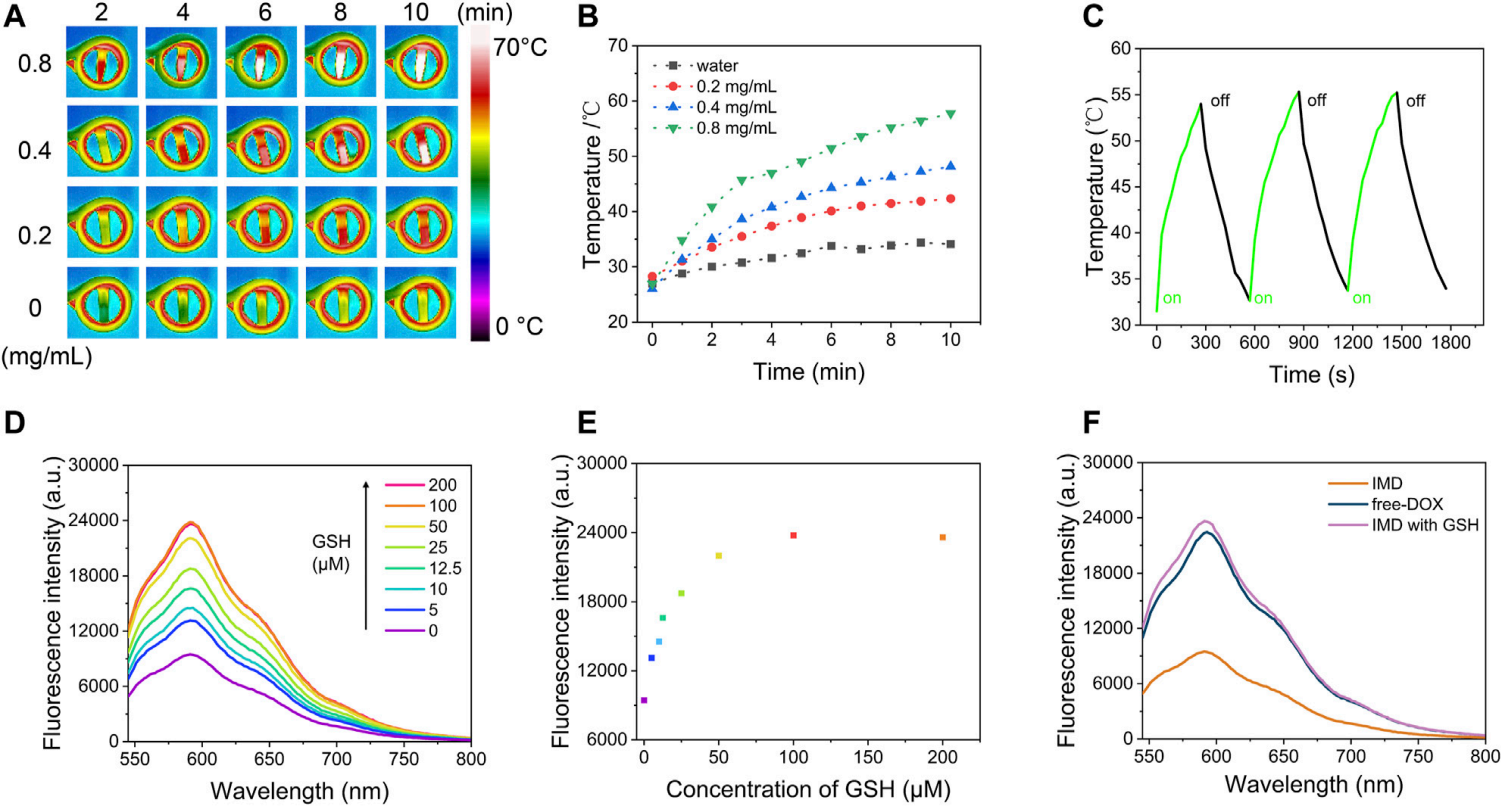
Magnetic hyperthermia performance and GSH-responsive properties of IMD. **(A)** Infrared thermal imaging images and **(B)** temperature increase curves of IMD (0, 0.2, 0.4, and 0.8 mg/ml) under an alternative magnetic field (power = 6 kW, frequency f = 474 kHz, and coil diameter = 3.5 cm) for 10 min. **(C)** The temperature change of IMD ([IO]: 0.8 mg/ml) under AMF stimulation (power = 6 kW, frequency f = 474 kHz) for 5 min, then AMF was turned off for 5 min, and this process was repeated three times. **(D)** Fluorescence spectra of IMD ([IO]: 0.16 mg/ml) incubated with GSH (0, 5, 10, 12.5, 25, 50, 100, and 200 μM) and **(E)** corresponding fluorescence intensity at 593 nm. **(F)** Fluorescence spectra of the free-DOX, IMD, and IMD with GSH. ([DOX]: 16 μg/ml).

To evaluate the magnetic hyperthermia capacity of IMD, different concentrations of IMD were placed in an AMF with a frequency of 474 kHz and current of 37.4 A. Figures 2A,B shows that after being exposed to AMF for 10 min, the temperature of IMD ([IO]: 0.8 mg/ml) increased from 26.93 to 57.78°C, while a slight change was observed in the control group (from 27.02 to 34.11°C). The temperature of IMD reached 48.16°C at 0.4 mg/ml, which was higher than the effective hyperthermia therapy temperature (>43°C) (Hervault and Thanh 2014). Simultaneously, we tested the magnetic hyperthermia stability of IMD by switching the AMF on and off for three cycles. Figure 2C shows that the temperature-increasing profile remained stable during the tests. These results indicate that IMD had excellent magnetic hyperthermia properties and could be used for the subsequent treatment.

### 3.3 Tumor Microenvironment-Responsive IMD Degradation

MnO_2_ is an efficient broad-spectrum fluorescence quencher because of its intense and broad optical absorption spectrum (∼200–600 nm) (Fan et al., 2015). But the fluorescence quenching ability is disabled upon its degradation to MnO_2_ and the release of loaded drugs. Figures 2D–F shows that the adsorption capacity of IMD for DOX by fluorescence analysis in different conditions. First, IMD ([IO]: 0.16 mg/ml) incubated with different concentrations of GSH (0, 5, 10, 12.5, 25, 50, 100, and 200 μM), and the fluorescence spectra of DOX were obtained after reaction for 12 min. MnO_2_ exhibited high quenching efficiency on DOX, and the fluorescence from DOX was completely quenched once it formed nanoparticles with MnO_2_. After, the fluorescence of DOX gradually recovered with increasing GSH concentration indicating MnO_2_ degradation and accompanied by a partial release of DOX. These results suggest that the increased release rate of DOX in drug release experiments is related to the structural damage of MnO_2_. It should be noted that the pH in normal tissue and blood is about 7.4, while it is weakly acidic in TME (Danhier et al., 2010). The above results suggested that IMD could respond to the TME, release DOX in the tumor area, and reduce the adverse effects of chemotherapeutic drugs caused by uncontrolled drug release.

### 3.4 Glutathione-Responsive MR/PA Imaging Capacity of IMD

**FIGURE 3:**
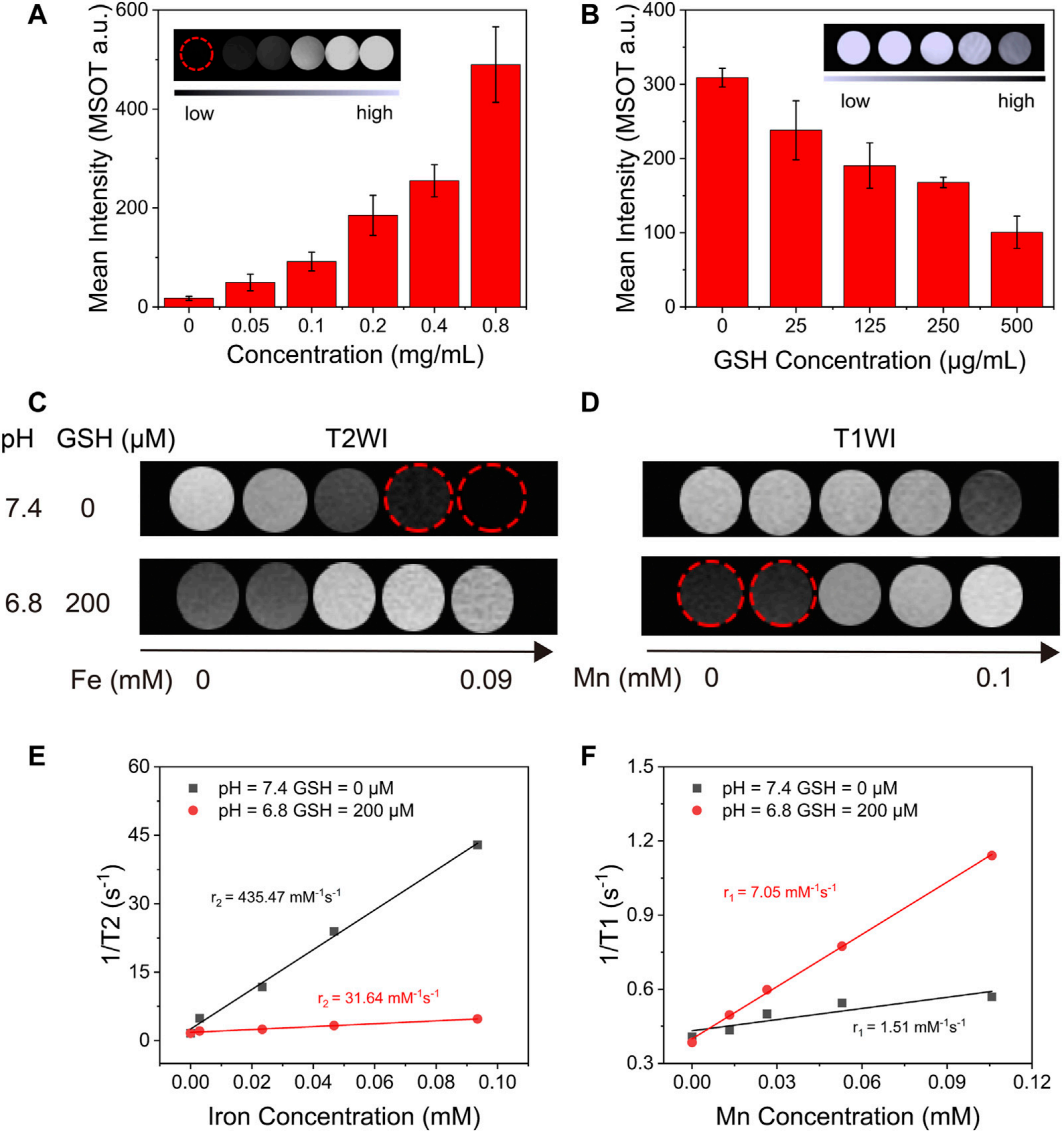
The *in vitro* TME-responsive PA/MR imaging. **(A)** The average intensity of PA imaging signals of IMD with different concentrations of IO (0–0.8 mg/ml). The inset shows the PA images of IMD at different concentrations of IO (0–0.8 mg/ml). **(B)** Average intensity of PA imaging signals of IMD ([IO]: 0.4 mg/ml) mixed with GSH (0, 25, 125, 250, and 500 μg/ml). **(C)** T2-and **(D)** T1-weighted MR images of IMD at different GSH concentrations and pH values. **(E)** The T2-weighted relaxivity (r2) and **(F)** T1-weighted relaxivity (r1) are calculated using the results of C and D.

A great deal of research shows that, as an excellent TME-responsive carrier, MnO_2_ could consume excessive GSH in solid tumors and release Mn^2+^, which could be used as a magnetic resonance (T1) contrast agent (Tian et al., 2019; Liu et al., 2021). Therefore, after evaluative the degradation of MnO_2_ with GSH, we intend to discuss the MR (T1) imaging capacity of IMD after degradation. Moreover, MnO_2_ has been reported as a PA agent at 750 nm (Liu et al., 2018). As MnO_2_ could be degraded with excessive GSH resulting in a convertible imaging effect, we investigated the potential of using IMD as a convertible MR/PA imaging agent. First, the PA imaging ability of IMD was evaluated. Figure 3A shows that the PA signal increased with IMD concentration, and the signal on the image changed from dark to bright. The signal strength showed a linear relationship with IMD concentration (Supplementary Figure S2). We also found that the PA signal showed a downward trend with the increased concentration of GSH because of the degradation of MnO_2_ (Figure 3B). The signal decline also showed a linear relationship with the GSH concentration (Supplementary Figure S3). Then we illustrated the responsive MR imaging ability of IMD on both T2-and T1-weighted imaging. Figures 3C–F shows that when without simulating TME, the T2 signal of IMD was enhanced and there was a shortened T2 relaxation time in T2-weighted MR imaging. However, there was no significant signal enhance change on T1-weighted imaging. After collecting images by quantitative magnetic resonance sequence T1 and T2 mapping, the ROI was plotted to obtain T1 and T2 relaxation times at different concentrations. Through linear fitting with the Mn and Fe concentration, r1 and r2 values were calculated as 1.51 mM^−1^s^−1^ and 435.47 mM^−1^s^−1^. However, when the TME was simulated *in vitro* (low pH, high GSH), the contrast effect of T2-weighted imaging was observed to significantly decrease and disappear with increasing GSH concentrations. In contrast, the enhanced effect of T1 was observed on T1-weighted imaging at this time. When the GSH increased to 200 μM and the pH value is 6.8, the r1 and r2 values were 7.05 mM^−1^s^−1^ and 31.64 mM^−1^s^−1^. The results showed that the enhanced effect of MR imaging T1 was significantly increased in the TME and in the presence of increased manganese ion concentrations, indicating that IMD was gradually degraded under mild acidic and abundant GSH conditions. These results indicate that the T1-weighted MR imaging performance is dependent on the cleavage of the MnO_2_ nanosheet. Moreover, the T2 effects changes may be related to the environment change of IO (from normal physiological conditions to TME). Because the presence of various molecules in the solution may prevent IO from accessing water molecules, thus affecting the T2 relaxation performance of IO. In addition, the aggregation state of IO may also play an important role in the T2 signal change of IMD under different conditions. Because in the TME condition, both the structure of IMD and the stability of the solution may change.

### 3.5 *In vitro* Cellular Uptake, Cytotoxicity, and Antitumor Effect

**FIGURE 4:**
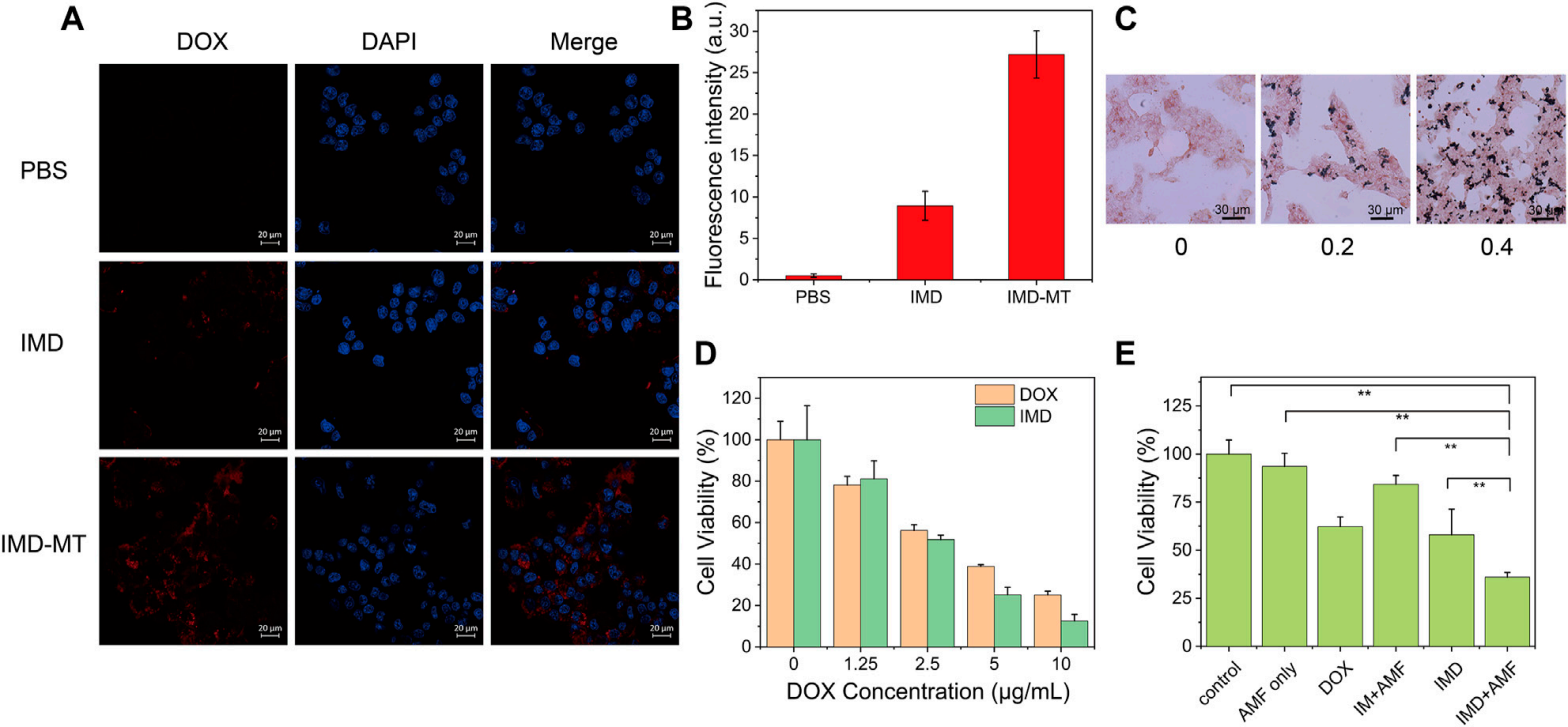
Cellular uptake and synergistic therapy efficacy of IMD. **(A)** Confocal images and **(B)** intracellular DOX fluorescence intensities of 4T1 cells incubated with IMD ([DOX]: 20 μg/ml) with or without magnetic targeting (MT). Scale bar: 20 μm. **(C)** Prussian blue staining of 4T-1 cells treated with different concentrations of IMD ([IO]: 0, 0.2, 0.4 mg/ml) for 6 h. **(D)** Cell viability of 4T1 cells treated with DOX and IMD for 24 h. **(E)** Cell viability of 4T1 cells treated with control, AMF only, DOX, IM + AMF, IMD and IMD + AMF ([DOX]: 10 μg/ml, [IO]: 0.1 mg/ml) for 12 h.

Magnetic targeting properties of IMD were observed in Supplementary Figure S4. After placing a magnet next to the IMD for 9 h, the IMD showed effective aggregation, indicating that IMD has magnetic targeting. Laser scanning confocal microscopy was used to monitor the cellular uptake of IMD based on the DOX fluorescence. 4T1 cells were incubated with IMD ([DOX]: 20 μg/ml) for 6 h in the presence or absence of a magnetic field. Figure 4A shows that compared with the IMD group, the fluorescence intensity in the magnetic targeting group was significantly more robust, which was consistent with the quantified fluorescence intensities of DOX in Figure 4B. The results showed that under the action of magnetic targeting, cells could absorb more IMD. Furthermore, the cellular uptake of IMD was also confirmed by Prussian blue staining (Figure 4C). The Prussian blue staining deepened with the increase of IMD concentration.

By MTT assay, we tested the cell inhibitory of free DOX and IMD with the same DOX concentration (0, 1.25, 2.5, 5, and 10 μg/ml). 4T1 cells were treated with free DOX or equivalent IMD and incubated for 12 and 24 h. Figure 4D and Supplementary Figure S5 show that the cytotoxicity of DOX and IMD were almost the same, proving that DOX-loaded IMD would retain its chemotherapeutic efficacy.

The cytotoxicity of the prepared IM without AMF condition was evaluated before we tested the synergistic therapeutic efficacy (Supplementary Figure S6). The MTT experiment showed negligible cytotoxicity for IM on 4T-1 cells even at the highest concentration ([IO]: 0.1 mg/ml), demonstrating the excellent biocompatibility of IM at this time. Next, we investigated the efficacy of IMD in combined magnetic hyperthermia and chemotherapy. 4T1 cells were co-incubated with RPMI-1640, RPMI-1640 with AMF (AMF only), DOX, IMD, IM with AMF (IM + AMF), or IMD with AMF (IMD + AMF) for 12 h, respectively. The IO and DOX concentrations (in all groups) were fixed at 100 μg/ml and 10 μg/ml. To improve the internalization of nanoparticles, a magnet was placed under the culture dishes for 4 h. The AMF only, IM with AMF, and IMD with AMF groups were exposed to AMF for 20 min (frequency 474 kHz, current 34.7 A). Figure 4E shows that the viability of 4T1 cells was 100, 93.57, 62.24, 84.22, 58.03, and 36.01% in the control, AMF only, free DOX, IM + AMF, IMD, and IMD + AMF, respectively. The results show no difference between the control and AMF groups, indicating that the magnetic field alone could not cause additional damage to cancer cells. Compared to other groups, the cell viability decreased significantly in IMD + AMF group, indicating that magnetic hyperthermia combined chemotherapy has a noticeable synergistic therapeutic effect.

### 3.6 *In vivo* Imaging

**FIGURE 5:**
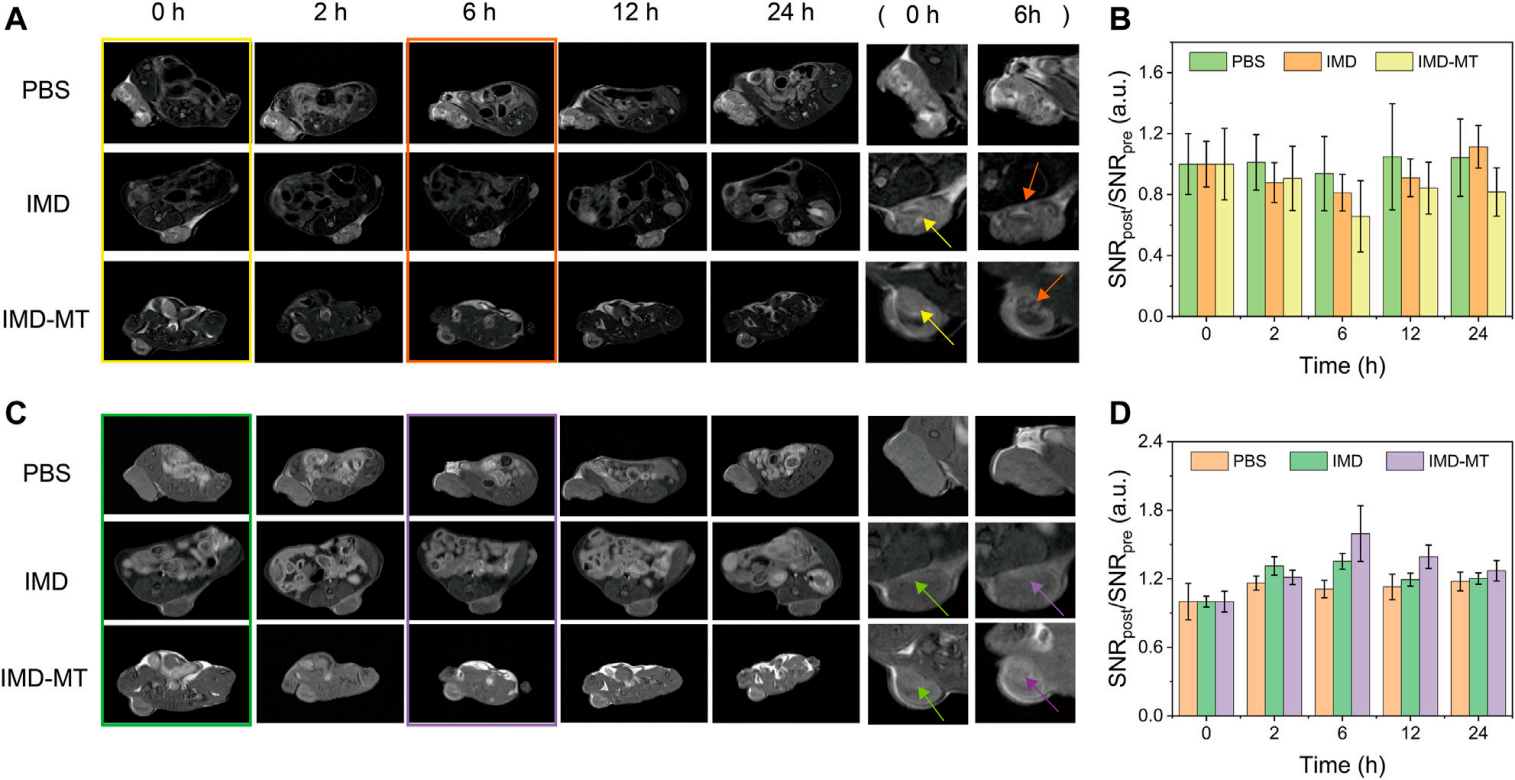
*In vivo* MR Imaging. **(A)** T2-weighted MR images of tumor-bearing mice at 0, 2, 6, 12, and 24 h after intravenous tail injection of IMD (DOX: 1 mg/kg, IO: 10 mg/kg). **(B)** Quantification of the T2 signal changes at the corresponding time points. **(C)** T1-weighted MR images of tumor-bearing mice at 0, 2, 6, 12, and 24 h after the intravenous tail injection of IMD. **(D)** Quantification of T1 signal changes at the corresponding time points.

Based on the bimodal PA and MR imaging performances of IMD *in vitro*, we further investigated the tumor diagnosis effect of IMD *in vivo*. 4T1 tumor-bearing mice were intravenously injected with the IMD (IO: 10 mg/kg), and MR/PA imaging images of tumor region were acquired at pre-designed time points (0, 2, 6, 12, and 24 h) to monitor the IMD accumulation in the tumor region. Mice were randomly divided into three groups: blank control, IMD, and IMD with magnetic targeting group. Figures 5A,B show that IMD illustrated a negative contrast enhancement effect and produced a low signal in MR imaging T2-weighted images at 6 h post-injection, especially in IMD with the magnetic-targeting group. Meanwhile, a bright signal in MR imaging T1-weighted images was also observed because of the release of Mn^2+^ caused by IMD degradation in the TME (Figures 5C,D). Moreover, compared with the control and IMD groups, the IMD with magnetic targeting group seemed to have more drug accumulation, which may be related to the synergism between the enhanced permeability and retention (EPR) effect and the magnetic targeting effect.

**FIGURE 6:**
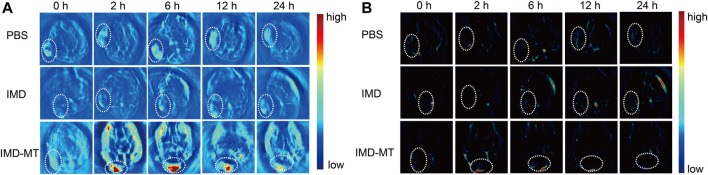
*In vivo* PA imaging. **(A)** The PA imaging and **(B)** multispectral optoacoustic tomography imaging of 4T-1 tumor-bearing mice at 0, 2, 6, 12, and 24 h after intravenous tail injection of IMD (DOX: 1 mg/kg, IO: 10 mg/kg).

We also performed PA imaging experiments to confirm the IMD accumulation and degradation in the tumor area. The results obtained in PA imaging were consistent with that in MR imaging (Figure 6A). As expected, significant signal enhancement was observed in the magnetic targeting group peaking at 6 h post-injection compared with the other groups. The signal remained relatively high within 24 h, demonstrating that IMD could realize effective drug accumulation and retention in the tumor area. Furthermore, as demonstrated previously, IMD could be degraded to release Mn^2+^, IO, and DOX in response to the tumor environment after accumulation. This process may be accompanied by the improvement of hypoxic conditions in the TME. To verify this procedure, we used the MSOT system to monitor the oxygenated hemoglobin (HbO_2_) concentration in real-time and evaluate whether magnetically targeted IMD could improve tumor hypoxia. After intravenous injection with IMD, the pseudo-color HbO_2_ images were obtained at pre-designed time points (0, 2, 6, 12, and 24 h). Figure 6B shows that the HbO_2_ signal intensity in the tumor site gradually increased in the magnetic-targeting IMD group, while in mice treated with PBS or IMD alone, negligible signal enhancement was observed. These observations suggested that oxygen may be released during IMD degradation, and that the released oxygen was trapped in the deoxyhemoglobin in the oxygen-containing hemoglobin within the erythrocyte (Yang et al., 2019).

### 3.7 *In vivo* Tumor Therapy

**FIGURE 7:**
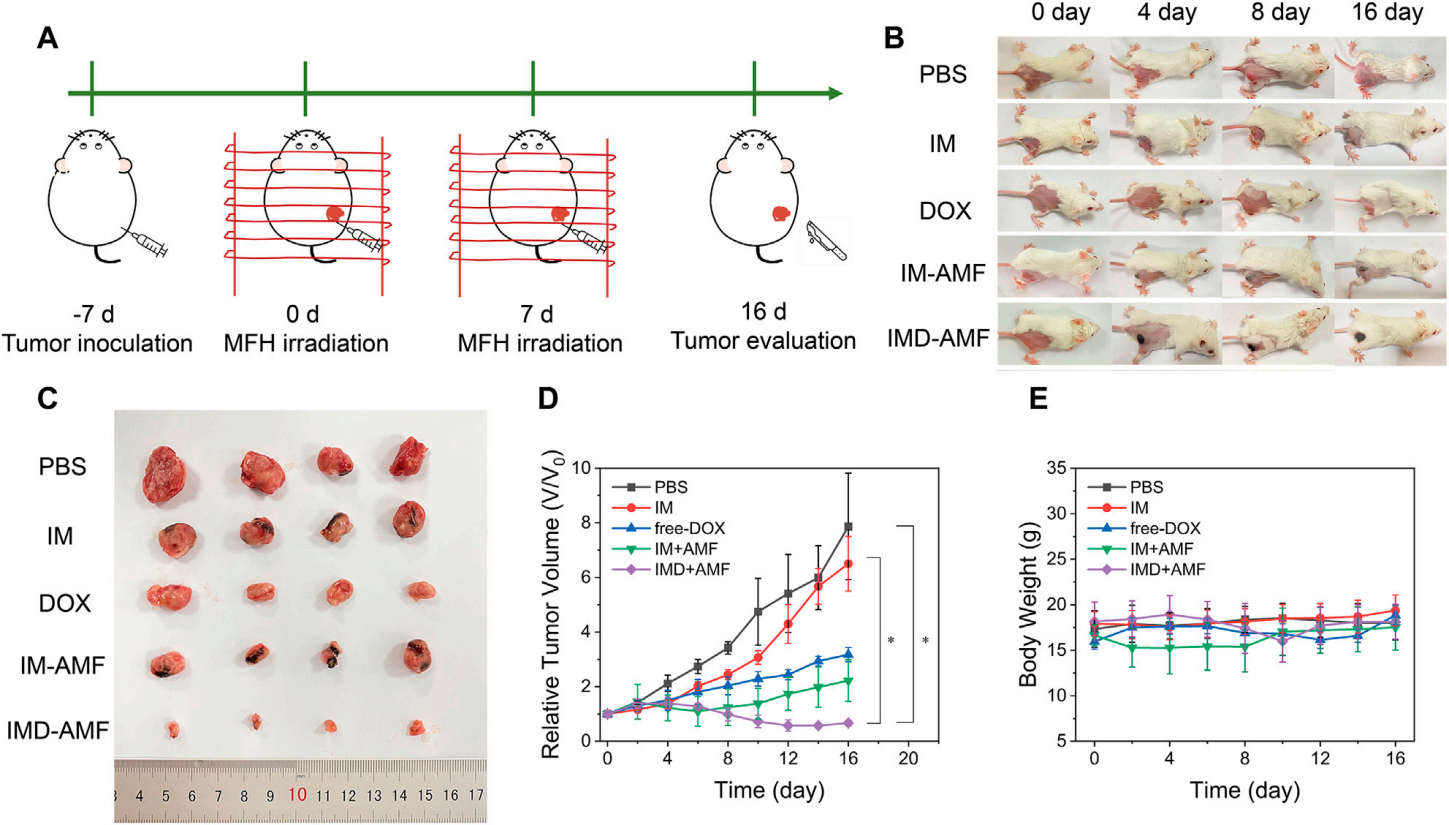
*In vivo* anticancer therapy. **(A)**. Schematic illustration of treatment schedules for *in vivo* antitumor therapy. **(B)** Photographs of the tumors were collected from different groups of mice at the end of treatment (days 0, 4, 8, and 16). **(C)** The photographs of eventual tumors of various treatments with intratumor injection. **(D)** Tumor growth curves and **(E)** body weight changes of different groups after various treatments.

Chemotherapy alone usually has many limitations, including low bioavailability and multiple side effects resulting from the non-selectivity of the drugs.(Lohitesh et al., 2018). Because magnetic nanoparticles have the ability of targeted drug delivery and have the effect of magnetocaloric therapy, magnetic nanoparticles combined with chemotherapeutic drugs can better achieve the effective treatment of cancer. In addition, intratumoral injection seems to achieve better local hyperthermia while reducing the risk of death from hyperthermia and potential systemic toxic and side effects after systemic administration. More importantly, intratumoral injection appears to be easier to achieve clinical transformation with magnetic hyperthermia. Here, we evaluated the potential of IMD for tumor thermo-chemotherapy after intratumoral injection. Before evaluating the efficacy of *in vivo* combination therapy, we have evaluated the tumor temperature of mice in the PBS + AMF group and the IMD + AMF group. An infrared thermal imager recorded the tumor temperature of the two groups of mice after 5 min of the alternating magnetic field. The results are shown in Supplementary Figure S7. The tumor temperature rises of the PBS + AMF group reached 34.9°C within 5 min. In comparison, the temperature rises of the IMD + AMF group reached 43.5°C, indicating excellent magnetic hyperthermia efficacy is expected to be achieved at this concentration dose, and AMF did not cause overheating in mice. To this end, the 4T-1 tumor-bearing mice were randomly divided into five groups as follows: 1) PBS, 2) IM, 3) DOX, 4) IM + AMF, and 5) IMD + AMF. The treatment plan was shown in Figure 7A. Tumor-bearing mice received treatment on days 0 and 7. Subsequently, the antitumor effect of each group was evaluated by monitoring the tumor volume every 2 days over 16 days Figures 7B,D revealed that except for the PBS and IM groups, the other groups showed a significant trend of tumor shrinkage after treatment. Additionally, after 16 days of treatment, mice in all groups were sacrificed, and then the tumor tissues were removed and photographed (Figure 7C). The tumors in the PBS and IM groups showed similar continuous tumor growth trends, with no significant difference in growth inhibition. The relative tumor volumes of the free DOX and IM + AMF groups increased slowly, indicating their inhibitory effect on tumor growth. IMD + AMF treatment exhibited the most significant delays on tumor growth with about a 40% reduction resulting from the synergistic effect of thermo-chemotherapy. Meanwhile, H&E staining of tumor tissue sections further confirmed its antitumor effect (Supplementary Figure S8). The tumor structure was not damaged and remained intact in the control group, while the tumor tissue of IMD + AMF mice showed considerable necrotic and structural damage.

Additionally, no significant body weight loss was observed in all groups, indicating that mice in each group were well tolerated at this therapeutic dose (Figure 7E). H&E staining also showed no visible organ damage indicating good biocompatibility of IMD (Supplementary Figure S9). It is worth discussing that putting a magnet on the tumor after intratumor administration seems to be a method worthy of consideration to achieve low-dose effective treatment because magnetic targeting prevents drug diffusion and indirectly improves the effectiveness of treatment (Xie et al., 2020).

## 4 Conclusion

In summary, we successfully prepared superparamagnetic iron oxide by thermal decomposition and generated a hybrid nanomaterial IMD by *in-situ* growth of manganese dioxide and electrostatic adsorption of DOX. IMD has the characteristics of magnetic targeting, tumor microenvironment response and allows for specific drug delivery. Moreover, IMD can be used as TME-responsive multimodal imaging (PA and MR T1-T2 double-contrast) agent, making it a promising PA/MR imaging contrast agent. Furthermore, the introduction of magnetic heat enhanced the anticancer activity of DOX *in vitro* and *in vivo* and effectively inhibited tumor growth. Besides, its magnetic targeting properties can better help nano drugs reach the tumor site. In the future, a major direction of magnetic hyperthermia is to use lower doses of drugs to achieve a more efficient treatment effect, so the design of nanoprobe maybe need to consider having more effective specific targeting ability and better magnetic thermal heating characteristics.

## Data Availability

The original contributions presented in the study are included in the article/Supplementary Material, further inquiries can be directed to the corresponding authors.
