# Supplementary material for: Developing Smart Nanoparticles Responsive to the Tumor Micro-Environment for Enhanced Synergism of Thermo-Chemotherapy With PA/MR Bimodal Imaging
Source: Front Bioeng Biotechnol. 2022 Feb 21;10:799610. doi: 10.3389/fbioe.2022.799610 (PMC8899915; doi:10.3389/fbioe.2022.799610)
Supplement: Supplementary file 1 [file DataSheet1.docx]

Supplementary Material


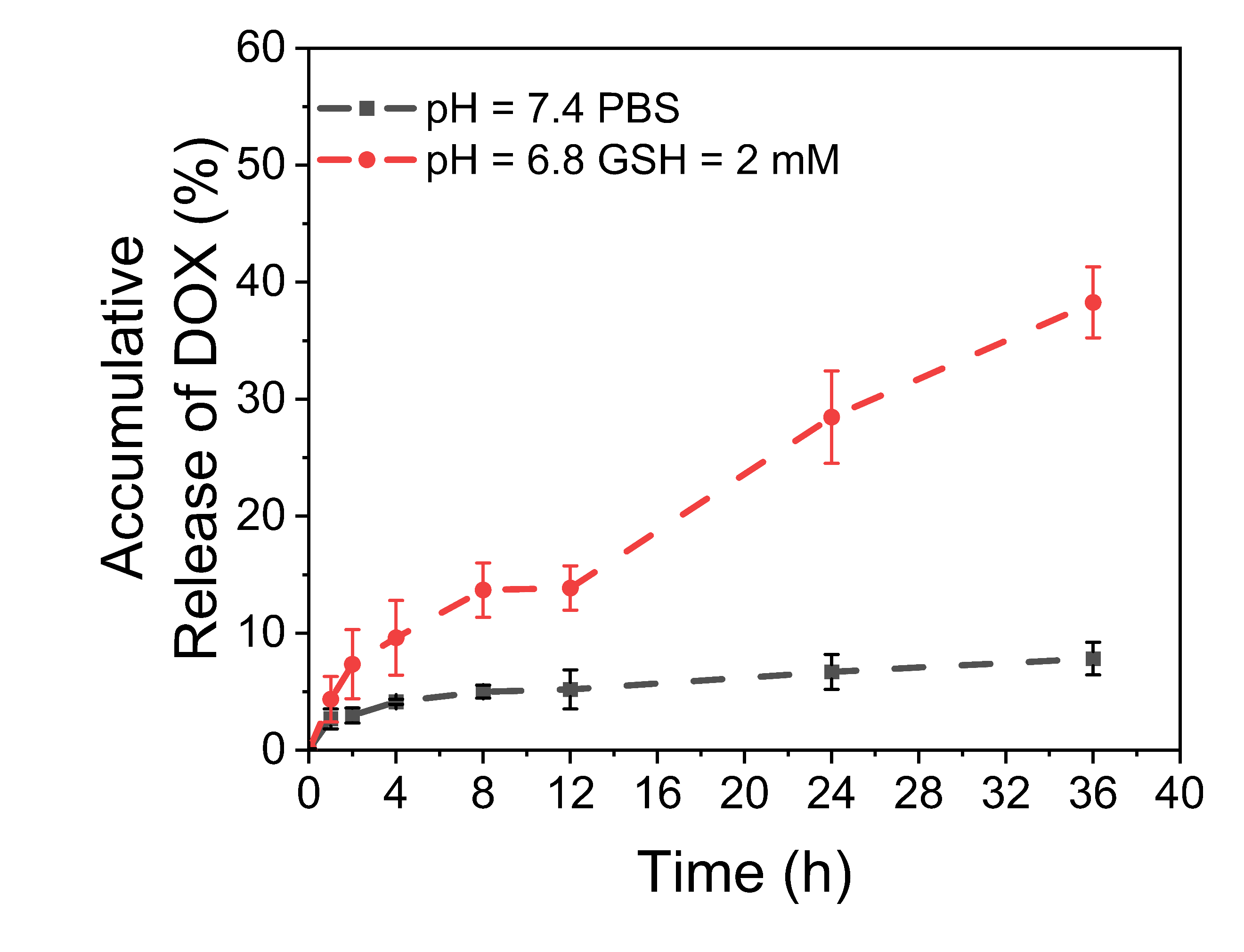


**Figure S1.** Accumulative DOX release from IMD in different environments.


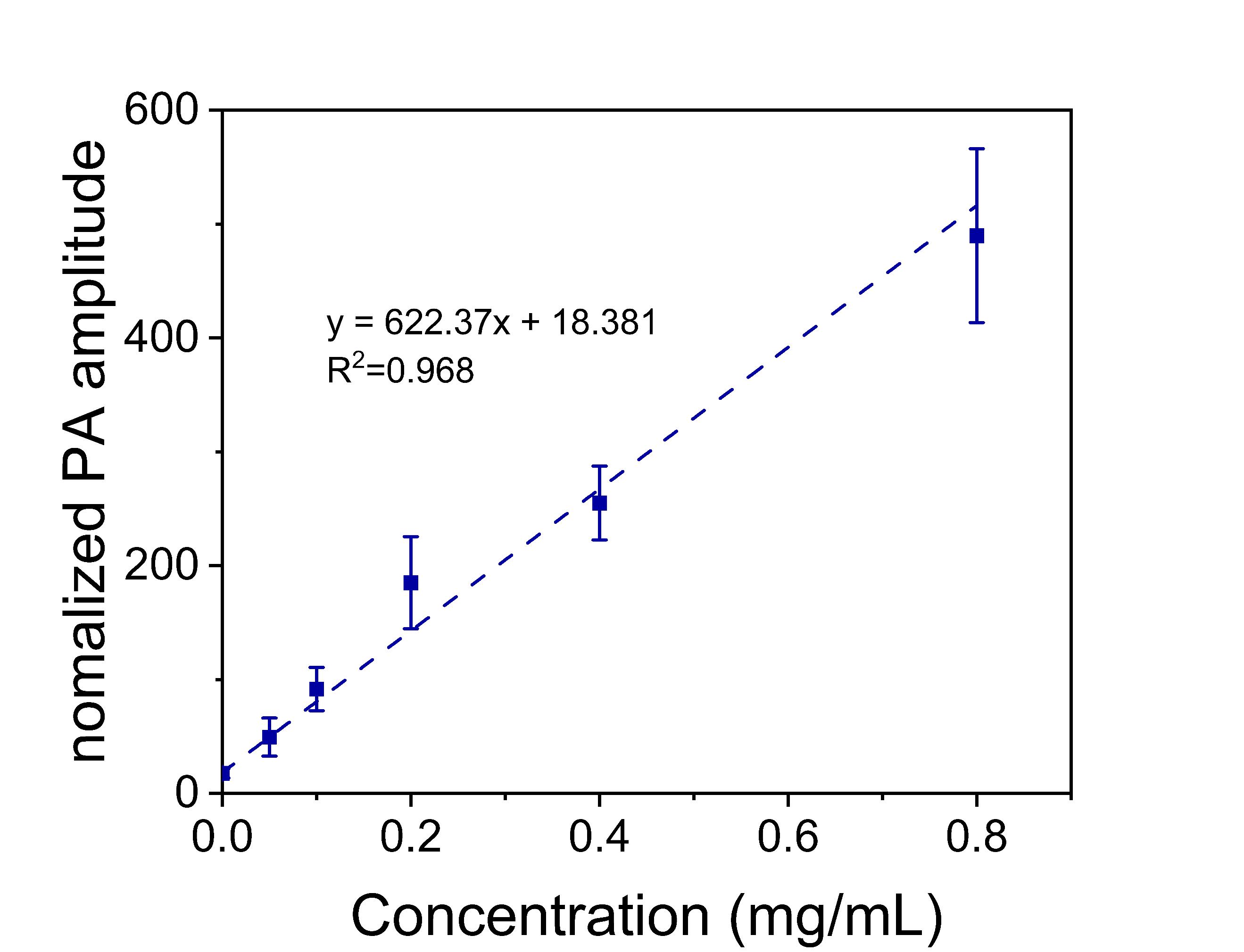


**Figure S2.** The linear relationship between IMD concentrations and normalized PA amplitudes for phantom tubes.


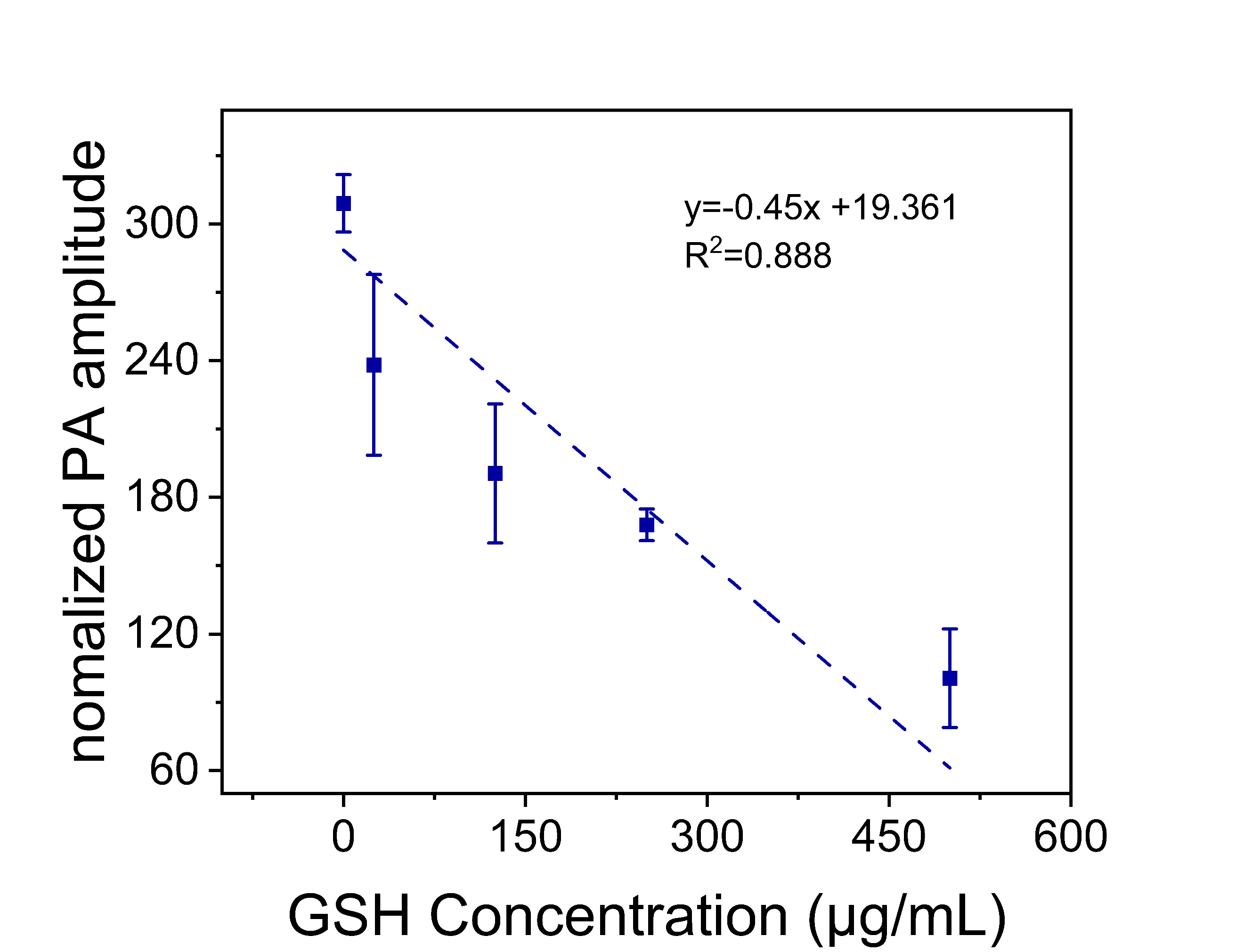


**Figure S3.** The linear relationship between GSH concentrations and normalized PA amplitudes for phantoms.


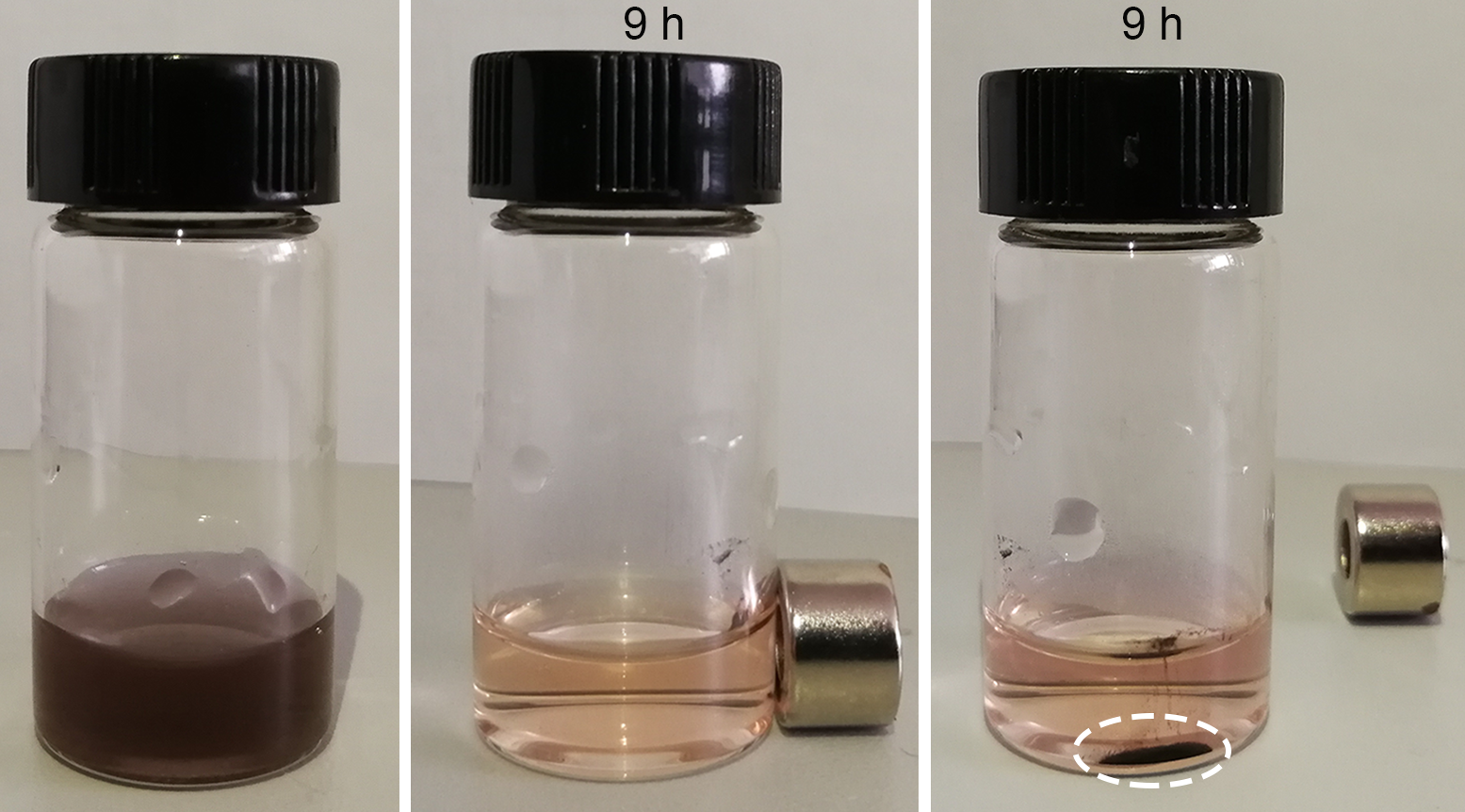


**Figure S4.** The magnetic targeting of IMD. The solution of IMD was placed near a magnet for 9 hours.


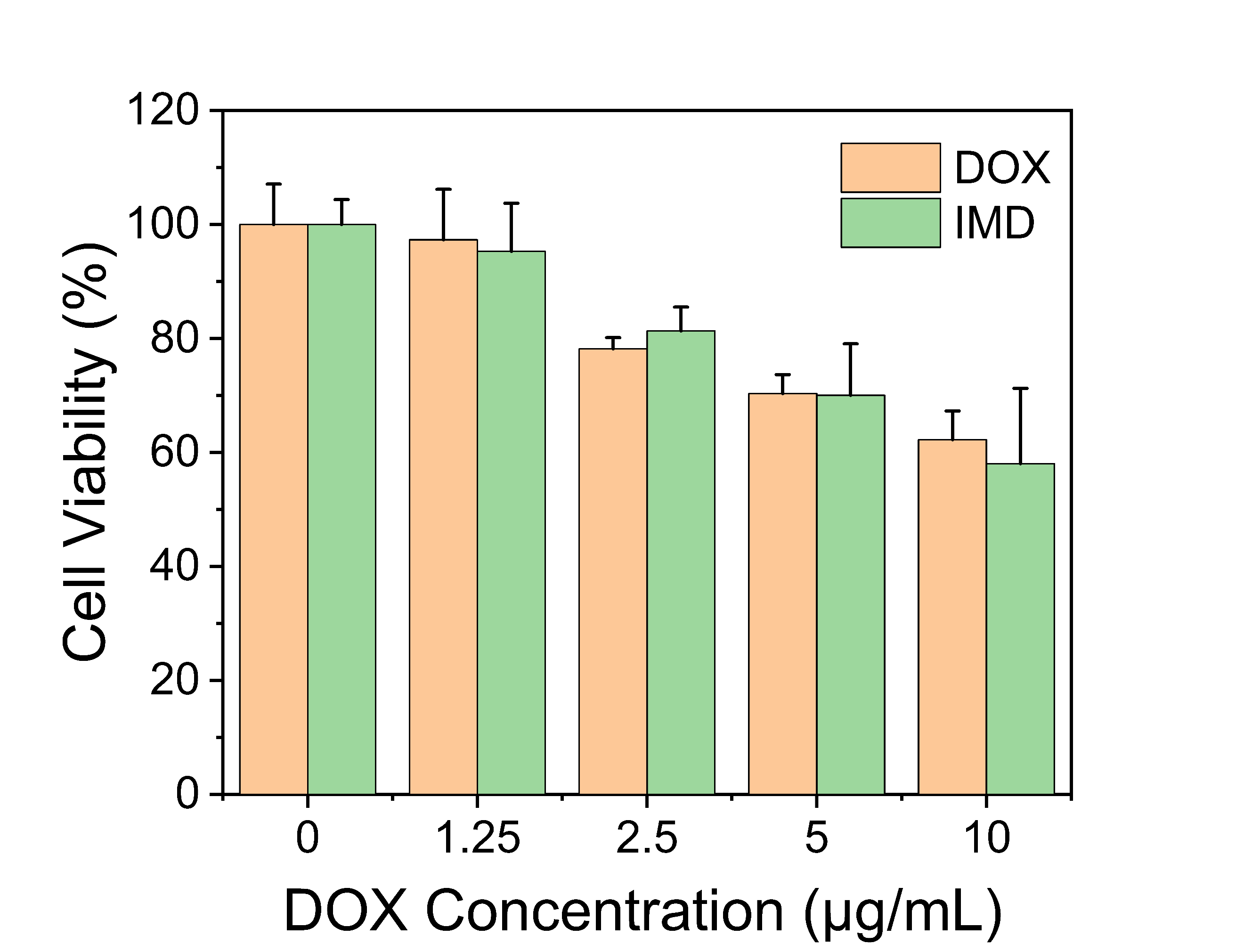


**Figure S5.** (D) Cell viability of 4T1 cells treated with DOX and IMD for 12 h.


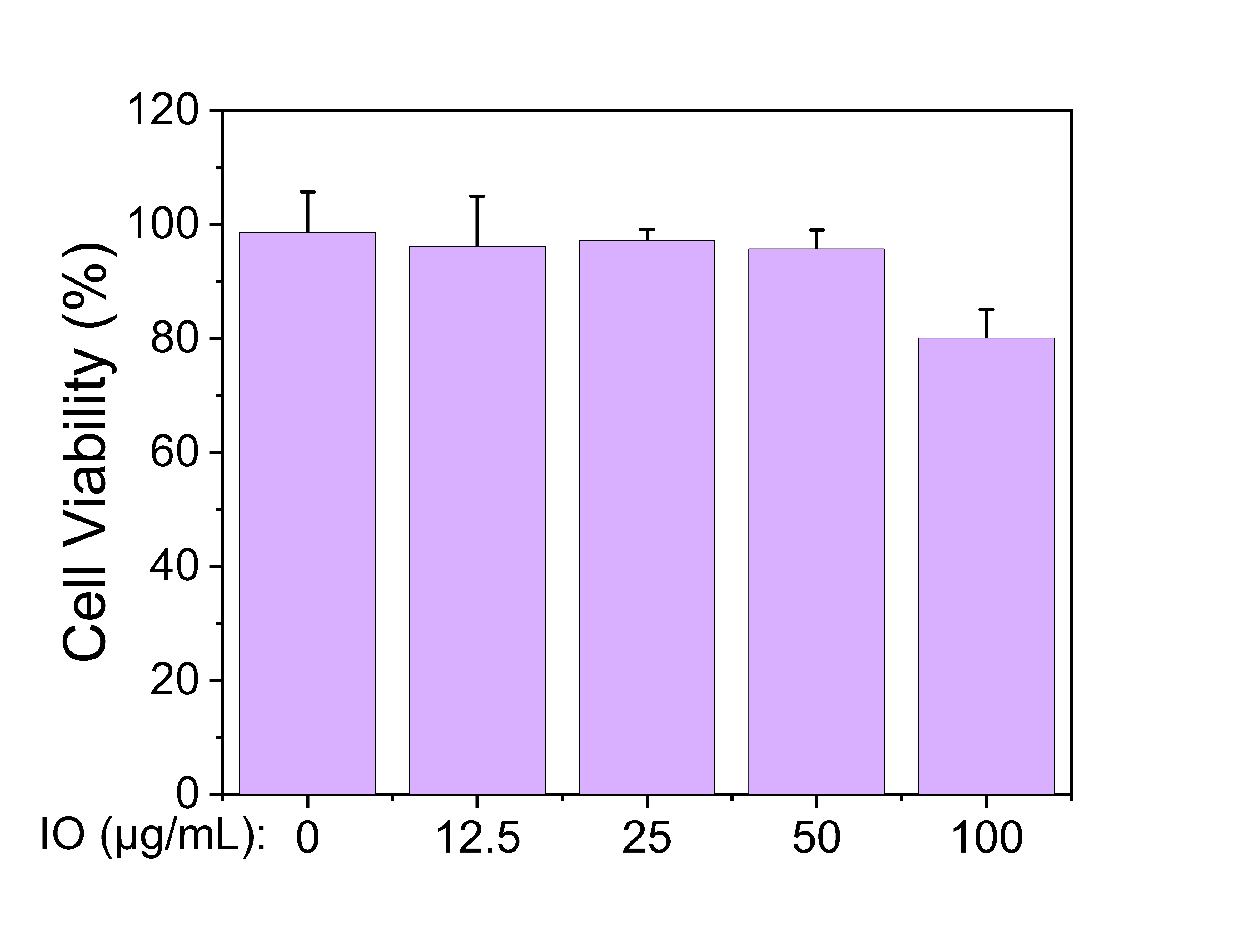


**Figure S6.** The cell viability of 4T1 cells treated with IM for 12 h.


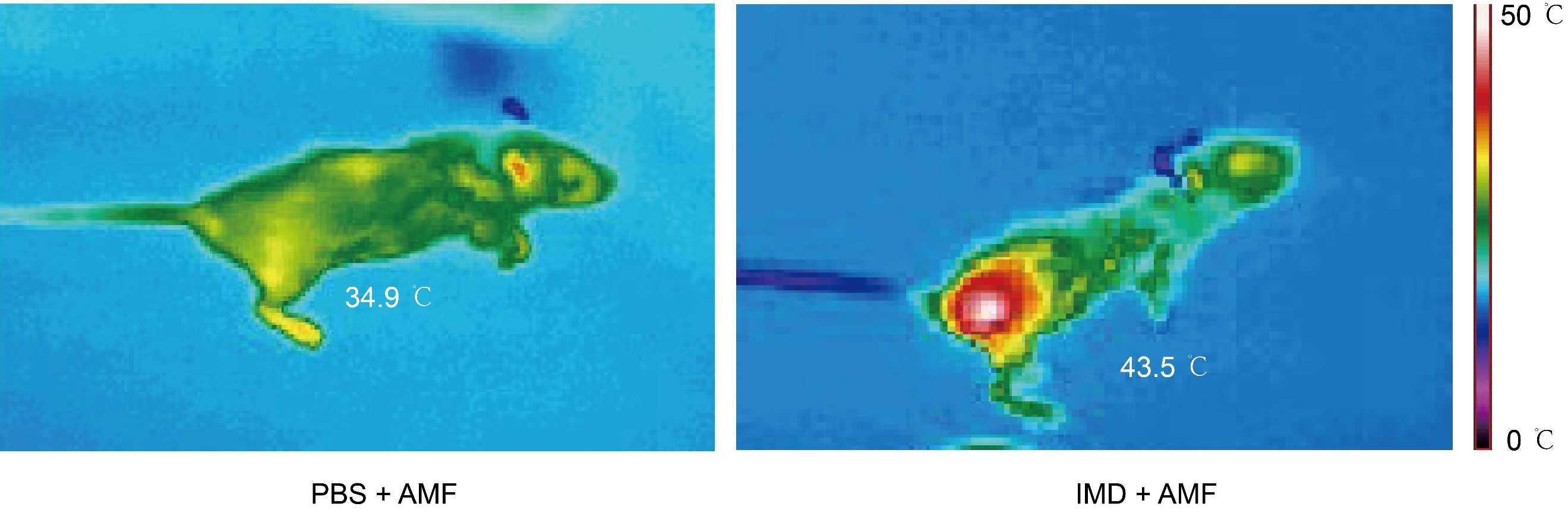


**Figure S7.** The infrared thermometer image shows the temperature of mice in the PBS group (left) and in the IMD group (right) under alternating magnetic field (AMF) for 5 min.


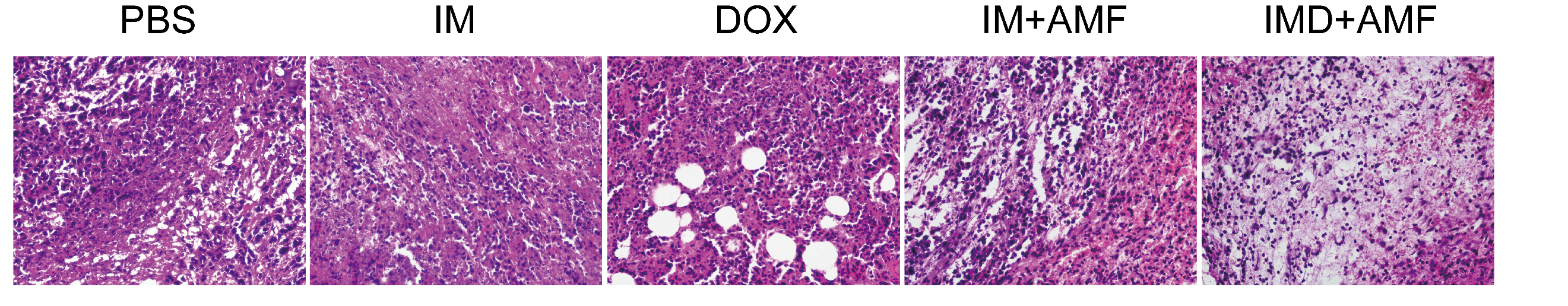


**Figure S8.** H&E staining of the tumor region 24 h after treatment (viewed at a magnification of 20×).


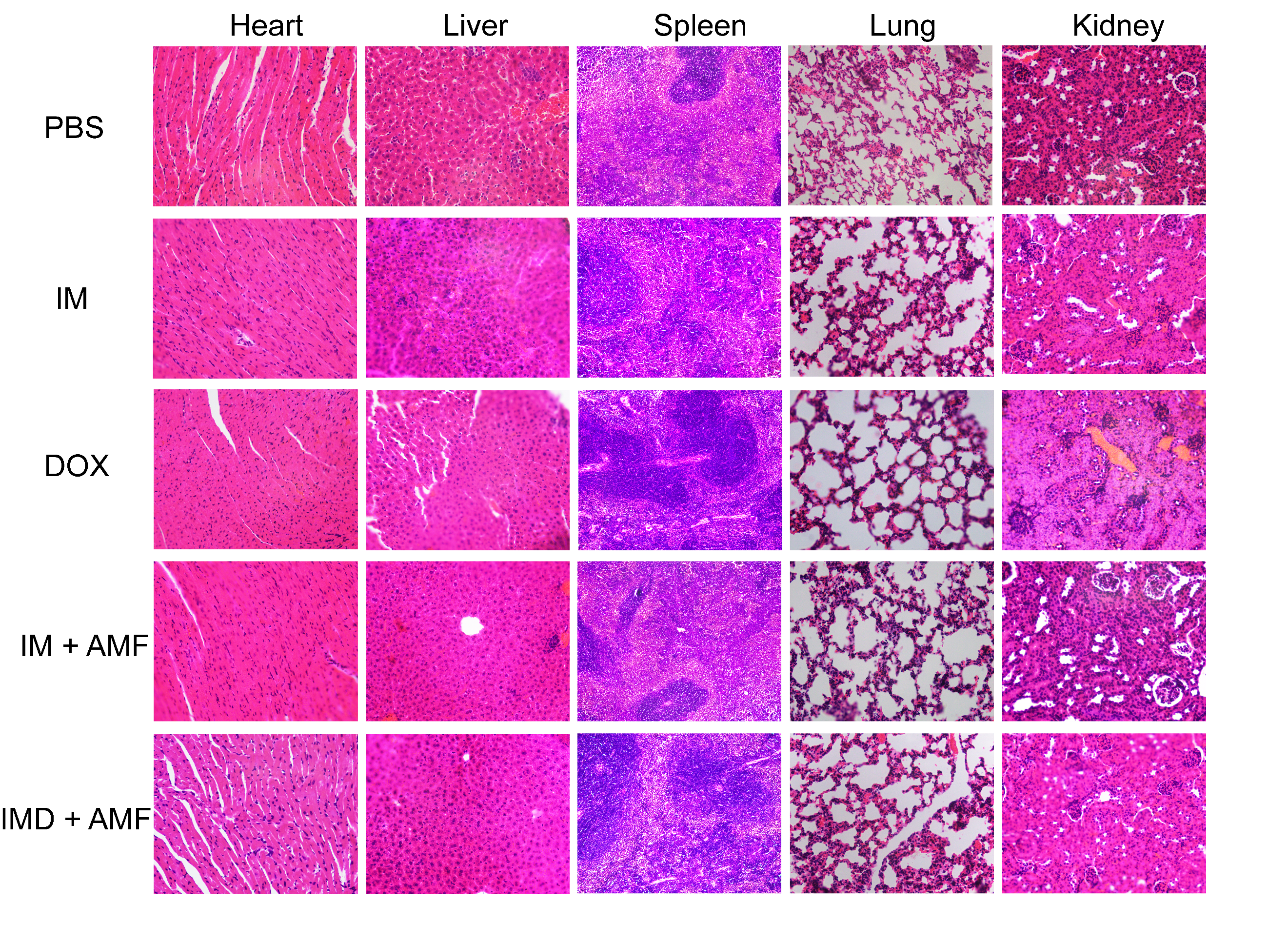


**Figure S9.** H&E staining of significant organs from mice treated with PBS, DOX, IM, IM with AMF, and IMD with AMF (viewed at a magnification of 20×).
